# The evolution of anesthesiology education: Embracing new technologies and teaching approaches

**DOI:** 10.1002/hsr2.1765

**Published:** 2024-01-29

**Authors:** Priska Bastola, Alok Atreya, Prawesh S. Bhandari, Subigya Parajuli

**Affiliations:** ^1^ Department of Cardiothoracic and Vascular Anaesthesiology, Manmohan Cardiothoracic and Vascular Center, Maharajgunj Medical Campus, Institute of Medicine Tribhuvan University Kathmandu Nepal; ^2^ Department of Forensic Medicine Lumbini Medical College Palpa Nepal; ^3^ Department of Orthopedics, Maharajgunj Medical Campus, Institute of Medicine Tribhuvan University Teaching Hospital, Tribhuvan University Kathmandu Nepal; ^4^ Research Program Coordinator, Department of Surgery and Perioperative Care The University of Texas Austin Texas USA

**Keywords:** medical education, teaching learning methods, web‐based tools

## Abstract

**Background and aims:**

Medical education requires regular reforms to include emerging best practices and technologies, while also critically evaluating effectiveness of traditional didactic teaching methods. This manuscript examines the challenges and opportunities associated with modernizing the anesthesiology curriculum.

**Methods:**

Narrative review of literature on innovations in medical education, with a specific emphasis on anesthesiology training.

**Results:**

Educators face difficulties implementing new teaching approaches and evaluating their effectiveness. However, active learning methods, blended with selected traditional techniques, can enhance learner engagement and competencies. Self‐directed learning and simulations prepare students for real‐world practice, while flipped classrooms and online platforms increase accessibility.

**Conclusions:**

A blended approach, integrating interactive technology alongside modified lectures and seminars, may optimize anesthesiology education. Despite the promise of improved pedagogies, further research is required to assess outcomes. By embracing innovation while retaining certain foundational methods, programs can equip anesthesiologists with modern skills. This evolution is key to meeting the needs of 21st‐century anesthesia care needs. Remaining at the forefront of this transformation will be vital in preparing competent future anesthesiologists through state‐of‐the‐art education.

## 1 INTRODUCTION

The field of medicine has undergone constant evolution since its beginning, creating challenges for those tasked with educating new generation physicians. Medical education aims to empower students by imparting the knowledge and skills necessary to thrive in their future profession. While teaching enables a person to acquire abilities through education and training,^1^ learning denotes the process of internal change in learner's attitudes, beliefs, behaviors, and knowledge.^2^ To keep pace with the rapid advances in medical technologies, drugs, and interventions, educators worldwide have sought optimal teaching approaches to enhance learning during student training. However, identifying the best methods to improve comprehension and retention remains an ongoing pursuit. Effective medical education requires educators to adapt their techniques and modalities to suit the evolving landscape of healthcare. This mandates increased research and innovation to refine pedagogical models and curriculum design. By embracing emerging best practices and technologies, while retaining select traditional approaches, medical schools can equip future physicians with the aptitudes required in a continuously transforming profession.

However, identifying the best methods to improve comprehension and retention remains an ongoing pursuit. Previously, programs focusing on increasing the competency of trainees adopted by the Royal College of Physicians and Surgeons^3^ were practiced, while later demands on the implementation of the “best evidence‐based medical system” in teaching and learning (BEME) gained popularity.^4^, ^5^

However, newer models based on competency designed and implemented in the training of medical trainees are gaining traction.^6^, ^7^ Anesthesiology education aims to impart the knowledge, skills, and critical thinking that trainees need to deliver safe, high‐quality perioperative care. Meeting this goal requires regular re‐evaluation of teaching practices as the field evolves.

Traditionally, pedagogy relied heavily on passive modalities such as didactic lectures and bedside demonstrations. However, these have limitations, including minimal learner engagement, inadequate development of practical competencies, and matching unique learning styles of the learners.^8^, ^9^, ^10^

This spurred efforts to implement more interactive, self‐directed approaches leveraging technology.^10^ E‐learning modules, virtual simulations, and game‐based learning are examples of such methods. They offer advantages such as self‐paced learning aligned with individual needs. However, evidence directly comparing learning outcomes from new versus traditional teaching is lacking. Their impact on clinical skill development also remains unclear.^11^ It is imperative that newer methods are rigorously evaluated as they are rapidly adopted worldwide. Understanding whether they are superior, equivalent, or inferior to traditional modalities will inform optimal instructional design.

This review critically analyzes anesthesiology teaching practices as they have evolved. This study explores the rationale for evaluating new modalities within limited existing evidence. The goal is to provide insights to help educators, institutions, and policymakers design curricula that best develop the next generation of competent anesthesiologists.

## 2 TEACHING AND LEARNING METHODS: PRESENT AND FUTURE

### 2.1 Present learning methods and tools

Current teaching practices in anesthesiology are increasingly incorporating technology to overcome the limitations of traditional teaching and learning approaches. The aim of educators has always been to design teaching techniques that provide a holistic learning experience for learners that incorporates theoretical as well as gaining practical skills.^12^ Taking into account the needs of the newer generation of web‐using students and their familiarity with the technology, which compelled the educators to accept the changing scenario and formulate newer educational modalities.^12^, ^13^ A gradual shift was seen from a more classroom‐based teaching and learning approach to outside classroom including online learning in anesthesiology programs with the goal of encouraging self‐directed learning.^14^ This was a challenge for the educators in anesthesiology as they had to initially learn for themselves what information technology is and its utility in designing teaching and learning methods to match the unique learning preferences of the new generation of learners.^12^ The adoption of online learning in anesthesiology came with its own challenges, including the need to inspire the educators to learn and adopt new technology, train the faculty members used to traditional teaching methods (textbooks, didactic lectures), to use technology for lectures (lecture podcasts), allot a separate budget for the endeavor, and develop a new system to assess the impact of e‐learning on the students.^12^, ^14^, ^15^ Newer tools currently used to enhance knowledge and skills of the anesthesiology trainees are simulators, digital learning platforms.

#### 2.1.1 Simulation‐based education

Simulation‐based training is another technological approach that has gained popularity in recent years since its inception nearly 50 years ago. Simulation training is an increasingly recognized pedagogical approach in anesthesia.^16^ It has been used to teach a variety of skills, from airway management to complex procedures requiring teamwork.^16^, ^17^ However, implementation of curricula requires substantial expertise, costs, and infrastructure.^18^ Future directions include expanding access through distributed networks and VR integration.

#### 2.1.2 Digital‐based education

Webinars, podcasts, and e‐Learning modules are examples of newer teaching methods that use technology to facilitate self‐directed and active learning. E‐learning modules allow self‐paced online learning with integrated quizzes, visuals, webinars, and podcasts make learning accessible outside the classroom.^12^ Although promising, robust evidence comparing these modalities to traditional teaching is lacking. Their impact on the development of clinical competencies also remains unclear.^19^, ^20^

### 2.2 Future learning methods and tools

Future immersive technologies such as virtual reality (VR) and augmented reality (AR) appear promising for enhancing anesthesiology education. Emerging VR platforms offer sophisticated three‐dimensional simulated operating room environments for rehearsing intricate anesthetic procedures. Evidence shows that recurrent hands‐on rehearsals in VR enhance the acquisition of technical skills compared with conventional training approaches.^21^ AR technology allows for simulated scenarios with embedded visual aids projected onto actual clinical settings to improve cognitive skills and situational decision‐making.^22^ As an example, AR could provide just‐in‐time visual cues during anesthesia administration simulations to boost learning. While self‐directed VR and AR platforms enable individual learning, traditional simulations remain superior for developing teamwork and communication competencies. Educators should strategically integrate VR and AR technologies based on available evidence to complement existing teaching methods and tailor training to desired learning objectives.^21^, ^22^ Although seeming beneficial, more comparative research is essential to confirm the actual impact of VR and AR and optimize their role in anesthesiology education.

### 2.3 Virtual game‐based learning

Game‐based learning incorporates gaming principles to actively engage learners, promote critical thinking, and build knowledge and skills through virtual experiences.^23^ In anesthesiology education, game simulations allow hands‐on practice in responding to challenges without expensive physical simulators. While promising for engagement, rigorous comparative research is needed to confirm game‐based learning's effectiveness over traditional teaching. Thoughtful integration based on evidence may optimize games as a supplemental modality, but more studies are essential to solidify it as an anesthesiology training approach.

### 2.4 Comparing past, present, and future practices

Till date, there has been an ongoing search for a comprehensive learning model to promote self‐directed learning among medical students and postgraduate anesthesia trainees.^12^, ^21^ Curricula have also transitioned from time‐based to competency‐based training, who demanded the use of a blended model of traditional as well as modern tools to promote holistic learning.^12^, ^14^, ^21^ Blended learning combines internet‐based tools with in‐person teaching to enhance learning.^12^ This approach personalizes digital education for residents with varying preferences and styles. It offers flexibility through modalities like podcasts, simulations, and game‐based learning, ensuring a customized experience. Studies directly comparing outcomes between new and old teaching methods are limited. Data indicates blended learning improves theoretical knowledge over classroom or e‐learning alone.^24^ However, gains in practical skills are often equivalent between blended and traditional approaches. Literature also reveals mixed impacts of e‐learning tools such as podcasts and videos on skills.^24^, ^25^ More rigorous comparative research is essential as new technologies are being rapidly adopted worldwide.

### 2.5 Anesthesia education in Nepal

The formal teaching of anesthesiology in Nepal began in 1985 with the launch of a 1‐year postgraduate diploma program by Tribhuvan University.^26^, ^27^ This program produced 46 anesthesiologists over 10 years. But recognizing the limitations of this short course, a 3‐year MD program was initiated in 1996 under the Postgraduate Medical Education Coordination Committee.^26^, ^28^

The aim was to develop an integrated curriculum to build skilled anesthesiologists. Canadian guidelines informed the initial model.^26^, ^27^ Despite limited resources, teaching emphasized books, lectures, and operating room discussions. Internet and mobile technology expanded pedagogical options.^29^ Simulation training was also introduced, initially in the Institute of Medicine and then in other centers.^26^

Over 20 years, competency‐based outputs have demonstrated progress. However, anesthesia education in Nepal still faces considerable challenges. Shortage of trained faculty, inadequate facilities and infrastructure, and limited fund hinders the quality and efficiency.^30^ Recommended standards for simulation training, online platforms, and other modalities remain unmet.

Blended learning, which combines classroom and online activities, is now popular given resource limitations. However, further efforts are imperative to contextualize curricula, expand simulation and technology access, incentivize faculty development, and promote international collaborations. Robust competency assessments must accompany instructional improvements. Addressing existing limitations will require increased investments in educational infrastructure and technologies.

Partnerships between institutions can pool limited resources and expertise. The government can also institute policies to standardize anesthesia training and promote innovations such as simulation‐based curricula. Anesthesia societies have a key role to play through advocacy and providing training scholarships.^31^ Despite difficulties, gradual enhancements in teaching practices aim to uplift anesthesia education in Nepal and produce competent specialist clinicians.^32^ A concise overview of how teaching and learning methods in anesthesiology have evolved over time, highlighting their objectives and potential future developments, is shown in Table 1.

**Table 1:** Comparison of different teaching and learning practices in anesthesiology.

Teaching and learning approaches	Description	Examples
Traditional	Emphasizes passive learning and didactic lectures	Didactic lectures, case studies, bedside teaching
Online and digital age	Using technology and focusing on self‐directed and active learning	e‐Learning modules, webinars, podcasts, virtual simulation
Game‐based learning	Incorporate game mechanics as a tool for active learning	START anesthesia, interactive simulations, digital quizzes
Blended teaching	Combines traditional and online methods to enhance learning	Flipped classrooms, hybrid courses
Future	The current trend is toward more learner‐centered approaches	Personalized learning, adaptive learning, artificial intelligence

### 2.6 Assessment of the trainees

Combining traditional and new assessment methods provides a thorough evaluation of anesthesia trainees’ growth in knowledge, skills, and professional competencies^33^, ^34^, ^35^:

- *Conventional written and oral exams*: Assess core knowledge, but may be less effective for formative feedback versus hands‐on assessments.^33^, ^35^
- *Direct observation tools (e.g., DOPS, A‐CEX)*: Evaluate clinical skills, critical thinking, procedural abilities through real or simulated patients.^33^, ^34^
- *Objective structured clinical examination (OSCE)*: Enable scenario‐based evaluation of clinical reasoning, critical thinking, technical skills, teamwork, and communication.^33^, ^34^
- *Simulations*: Evaluate reasoning, critical thinking, technical skills, teamwork, and communication using sophisticated simulations.^33^, ^34^
- *Multisource feedback*: Gain insights on collaboration, communication, and professionalism from various sources.^33^, ^35^
- *Competency‐based assessments*: Matching training with gaining expertise in knowledge, procedures, and non‐technical areas.^33^

### 2.7 Anesthesia education in the digital age

Anesthesia education is undergoing a major digital transformations, presenting new opportunities as well as challenges.^36^, ^37^, ^38^, ^39^, ^40^ Online forums and social media allow networking, self‐learning, and global collaboration.^36^, ^37^ But they also need data security and professional conduct.^36^, ^37^ Artificial intelligence (AI) offers new learning capabilities^39^ but it must be done ethically to preserve learner autonomy and prevent bias.^36^, ^39^ Combining traditional skills training with new AI has the potential to improve anesthesia education.^40^ However, integration requires services, resources, faculty expertise,^36^ and a uniform and transparent approaches.^36^, ^37^, ^38^ The sudden shift to virtual education during COVID‐19 has highlighted this need worldwide.^41^ Emerging technologies have great potential to transform anesthesia education worldwide by considering integration while addressing ethical concerns.^36^, ^37^, ^38^, ^39^, ^40^

## 3 ETHICAL CONCERNS OF AI INTEGRATION

Using AI in anesthesia education raises important ethical issues. Policies for AI should be developed and implemented in a transparent manner and monitored for bias.^40^, ^42^ Patient confidentiality and autonomy must be protected,^40^ and the physician–patient relationship must be preserved. The development and use of AI should follow guidelines and policies,^42^, ^43^ including the adaptation to local contexts.^40^ Education on proper integration of AI is important.^42^ The complementary role of AI should be emphasized,^40^ as human clinical judgment remains paramount.

## 4 CONCLUSIONS

The shift toward active, self‐directed learning seems promising, but using new technologies effectively remains a challenge. Comprehensive assessment of knowledge, skills, and attitudes is vital to train competent anesthesiologists and ensure safe care. More rigorous research to compare new and traditional teaching methods, particularly on developing clinical skills, is essential. Carefully blending traditional techniques with innovations such as simulations, online platforms, and game‐based learning can optimize outcomes by tailoring learning to varying needs and styles of student. Further evaluation is needed to confirm the benefits and feasibility of VR and AR.

Assessing trainees through a multifaceted approach using traditional testing, simulations and advanced technologies can provide insight into their development in knowledge, skills, and professional domains. However, barriers to faculty of new assessment tools arise.

Efforts are being made to modernize anesthesia education curricula worldwide, including Nepal. However, difficulties in securing adequate resources, infrastructure, and faculty training hinder progress. Partnerships between institutions and support from governments and professional societies can help overcome these limitations.

By adapting to change while maintaining some core methods, anesthesiology programs can deliver high‐quality education to prepare physicians for 21st‐century practice. More research on the outcomes of emerging modalities will inform optimal instructional design. It is important to keep pace with developments to train future anesthesiologists capable of meeting the needs of the dynamic healthcare landscape.

## AUTHOR CONTRIBUTIONS

**Priska Bastola**: Conceptualization; supervision; writing—original draft; writing—review & editing. **Alok Atreya**: Supervision; writing—original draft; writing—review & editing. **Prawesh S. Bhandari**: Conceptualization; writing—original draft; writing—review & editing. **Subigya Parajuli**: Conceptualization; writing—original draft; writing—review & editing.

## CONFLICT OF INTEREST STATEMENT

The authors declare no conflict of interest.

## ETHICS STATEMENT

This review article did not involve any human participants or animals. Therefore, ethical approval was not required.

## TRANSPARENCY STATEMENT

The lead author Priska Bastola affirms that this manuscript is an honest, accurate, and transparent account of the study being reported; that no important aspects of the study have been omitted; and that any discrepancies from the study as planned (and, if relevant, registered) have been explained.

## Data Availability

The data analyzed in this review article were obtained from publicly available sources, including databases, academic journals, government publications, and reputable websites. All data used in this study are cited and referenced appropriately. No new data were collected for this review article.

## References

[1] Bisset A . The Canadian Oxford Paperback Dictionary. Oxford University Press Canada; 2000.

[2] Ambrose SA , ed. How Learning Works: Seven Research‐Based Principles for Smart Teaching . The Jossey‐Bass Higher and Adult Education Series. 1st ed. Jossey‐Bass; 2010:301.

[3] Frank JR , Danoff D . The CanMEDS initiative: implementing an outcomes‐based framework of physician competencies. Med Teach. 2007;29(7):642‐647. 18236250 10.1080/01421590701746983

[4] Harden M , Best PMLR . evidence medical education: the simple truth. Med Teach. 2000;22(2):117‐119.

[5] Hart Ian R. , Harden RM . Best evidence medical education (BEME): a plan for action. Med Teach. 2000;22(2):131‐135.

[6] Djulbegovic B , Guyatt GH . Progress in evidence‐based medicine: a quarter century on. Lancet. 2017;390(10092):415‐423. 28215660 10.1016/S0140-6736(16)31592-6

[7] Stodel EJ , Wyand A , Crooks S , Moffett S , Chiu M , Hudson CCC . Designing and implementing a competency‐based training program for anesthesiology residents at the University of Ottawa. Anesthesiol Res Pract. 2015;2015:1‐7. 10.1155/2015/713038 PMC4698531 26798337

[8] Nehete A , Sharma R , Reazaul Karim H , Rana S . Need to review anaesthesia curriculum and education!. Indian J Anaesth. 2022;66(1):87‐90. 35309026 10.4103/ija.ija_1105_21 PMC8929319

[9] Marchalot A , Dureuil B , Veber B , et al. Effectiveness of a blended learning course and flipped classroom in first year anaesthesia training. Anaesth Crit Care Pain Med. 2018;37(5):411‐415. 29175318 10.1016/j.accpm.2017.10.008

[10] University of Queensland, Blackburn G . Innovative elearning: Technology shaping contemporary problem based learning: a cross‐case analysis. J Univ Teach Learn Pract. 2015;12(2):51‐68.

[11] Ebert TJ , Fox CA . Competency‐based education in anesthesiology. Anesthesiology. 2014;120(1):24‐31. 24158052 10.1097/ALN.0000000000000039

[12] Kannan J , Kurup V . Blended learning in anesthesia education: current state and future model. Curr Opin Anaesthesiol. 2012;25(6):692‐698. 23147669 10.1097/ACO.0b013e32835a1c2a

[13] Kundra P , Kurdi M , Mehrotra S , Jahan N , Kiran S , Vadhanan P . Newer teaching‐learning methods and assessment modules in anaesthesia education. Indian J Anaesth. 2022;66(1):47‐57. 35309022 10.4103/ija.ija_1103_21 PMC8929315

[14] Kurup V , Hersey D . The changing landscape of anesthesia education: is Flipped Classroom the answer? Curr Opin Anaesthesiol. 2013;26(6):726‐731. 24126692 10.1097/ACO.0000000000000004

[15] Shangraw RE , Whitten CW . Managing intergenerational differences in academic anesthesiology. Curr Opin Anaesthesiol. 2007;20(6):558‐563. 17989549 10.1097/ACO.0b013e3282f132e3

[16] Sun Y , Pan C , Li T , Gan TJ . Airway management education: simulation based training versus non‐simulation based training—a systematic review and meta‐analyses. BMC Anesthesiol. 2017;17(1):17. 28143389 10.1186/s12871-017-0313-7 PMC5286685

[17] Yunoki K , Sakai T . The role of simulation training in anesthesiology resident education. J Anesth. 2018;32:425‐433. 29523995 10.1007/s00540-018-2483-y

[18] Byrick RJ , Naik VN , Wynands JE . Simulation‐based education in Canada: will anesthesia lead in the future? Can J Anesth J Can Anesth. 2009;56(4):273‐278. 10.1007/s12630-009-9053-6 19296188

[19] Bock A , Kniha K , Goloborodko E , et al. Effectiveness of face‐to‐face, blended and e‐learning in teaching the application of local anaesthesia: a randomised study. BMC Med Educ. 2021;21:137. 33639906 10.1186/s12909-021-02569-z PMC7913455

[20] Andersson ML , Duch P , Bessmann EL , Lundstrøm LH , Ekelund K . Preparing for obstetric anaesthesia—an educational randomised controlled trial comparing e‐learning to written course material. Acta Anaesthesiol Scand. 2023;67(1):36‐43. 36112027 10.1111/aas.14148 PMC10092133

[21] Alam F , Matava C . A new virtual world? The future of immersive environments in anesthesiology. Anesth Analg. 2022;135(2):230‐238. 35839493 10.1213/ANE.0000000000006118 PMC9259048

[22] Privorotskiy A , Garcia VA , Babbitt LE , Choi JE , Cata JP . Augmented reality in anesthesia, pain medicine and critical care: a narrative review. J Clin Monit Comput. 2022;36(1):33‐39. 33864581 10.1007/s10877-021-00705-0

[23] Chandrasoma J , Chu LF . Teaching the 21st century learner: innovative strategies and practical implementation. Int Anesthesiol Clin. 2016;54(3):35‐53. 27285071 10.1097/AIA.0000000000000108

[24] Bock A , Kniha K , Goloborodko E , et al. Effectiveness of face‐to‐face, blended and e‐learning in teaching the application of local anaesthesia: a randomised study. BMC Med Educ. 2021;21(1):137. 33639906 10.1186/s12909-021-02569-z PMC7913455

[25] Vallée A , Blacher J , Cariou A , Sorbets E . Blended learning compared to traditional learning in medical education: systematic review and meta‐analysis. J Med Internet Res. 2020;22(8):e16504. 32773378 10.2196/16504 PMC7445617

[26] Amatya R . Evolution of anesthesia in Nepal: a historical perspective. J Soc Anesthesiol Nepal. 2015;1(1):3‐6.

[27] Shrestha BM , Rana NB . Training and development of anesthesia in Nepal—1985 to 2005. Can J Anesth J Can Anesth. 2006;53(4):339‐343. 10.1007/BF03022496 16575030

[28] Karki DB , Dixit H . An overview of undergraduate and postgraduate medical education in Nepal and elsewhere. Kathmandu Univ Med J. 2004;2(1):69‐74. 19780293

[29] Shah S , Ross O , Pickering S . Distance‐learning initiatives targeting non‐physician anesthesia providers in low‐resource environments. Curr Anesthesiol Rep. 2021;11(1):64‐68. 33519304 10.1007/s40140-020-00428-z PMC7835316

[30] Neupane HC , Shrestha N , Shrestha BK . Fellowship training in Nepal: current prospects. J Nepal Health Res Council. 2018;16(3):345‐350. 30455498

[31] Nepal L . Anesthesia in Nepal: from history to current scenario. Anaesth Pain Intensive Care. 2019;23:102‐104.

[32] Shrestha A . Anaesthesia education in Nepal—what residents think. J Soc Anesthesiol Nepal. 2015;1(2):65‐69.

[33] Kundra P , Kurdi M , Mehrotra S , Jahan N , Kiran S , Vadhanan P . Newer teaching‐learning methods and assessment modules in anaesthesia education. Indian J Anaesth. 2022;66(1):47. 35309022 10.4103/ija.ija_1103_21 PMC8929315

[34] Training Requirements for the Specialty of Anaesthesiology. European Union of Medical Specialists. 2022. https://www.uems.eu/__data/assets/pdf_file/0004/156199/UEMS-2022.12-European-Training-Requirements-in-Anaesthesiology.pdf

[35] Weller JM , Naik VN , San Diego RJ . Systematic review and narrative synthesis of competency‐based medical education in anaesthesia. Br J Anaesth. 2020;124(6):748‐760. 32008702 10.1016/j.bja.2019.10.025

[36] Kearsley R , MacNamara C . Social media and online communities of practice in anesthesia education. Anaesthesia. 2019;74(9):1202‐1203. 31386189 10.1111/anae.14764

[37] Charlesworth M . Social media and online communities of practice in anaesthesia education–a reply. Anaesthesia. 2019;74(9):1203. 31386187 10.1111/anae.14765

[38] Kiran S , Sethi N . Anaesthesiologist and social media: walking the fine line. Indian J Anaesth. 2018;62(10):743. 30443055 10.4103/ija.IJA_449_18 PMC6190416

[39] Alowais SA , Alghamdi SS , Alsuhebany N , et al. Revolutionizing healthcare: the role of artificial intelligence in clinical practice. BMC Med Educ. 2023;23(1):689. 37740191 10.1186/s12909-023-04698-z PMC10517477

[40] Koçer Tulgar Y , Tulgar S , Güven Köse S , et al. Anesthesiologists’ perspective on the use of artificial intelligence in ultrasound‐guided regional anaesthesia in terms of medical ethics and medical education: a survey study. Eurasian J Med. 2023;55(2):146‐151. 37161553 10.5152/eurasianjmed.2023.22254 PMC10440966

[41] Atreya A , Acharya J . Distant virtual medical education during COVID‐19: half a loaf of bread. Clin Teach. 2020;17(4):418‐419. 32558269 10.1111/tct.13185 PMC7323172

[42] Cascella M , Tracey MC , Petrucci E , Bignami EG . Exploring artificial intelligence in anesthesia: a primer on ethics, and clinical applications. Surgeries. 2023;4(2):264‐274.

[43] Busch F , Adams LC , Bressem KK . Biomedical ethical aspects towards the implementation of artificial intelligence in medical education. Med Sci Educ. 2023;33(4):1007‐1012. 37546190 10.1007/s40670-023-01815-x PMC10403458

